# Pasteurization of human milk affects the miRNA cargo of EVs decreasing its immunomodulatory activity

**DOI:** 10.1038/s41598-023-37310-x

**Published:** 2023-06-21

**Authors:** Monica F. Torrez Lamberti, Leslie A. Parker, Claudio F. Gonzalez, Graciela L. Lorca

**Affiliations:** 1 Department of Microbiology and Cell Science, Genetics Institute, Institute of Food and Agricultural Sciences, University of Florida, Gainesville, USA; 2 College of Nursing, University of Florida, Gainesville, USA

**Keywords:** Cell signalling, Nutrition

## Abstract

In this report, we evaluated the effect of the pasteurization (P) process of mother’s own milk (MOM) on the miRNA content of extracellular vesicles (EVs) and its impact on innate immune responses. Differences in size or particle number were not observed upon pasteurization of MOM (PMOM). However, significant differences were observed in the EV membrane marker CD63 and miRNA profiles. miRNA sequencing identified 33 differentially enriched miRNAs between MOM_EV_ and PMOM_EV_. These changes correlated with significant decreases in the ability of PMOM_EV_ to modulate IL-8 secretion in intestinal Caco2 cells where only MOM_EV_ were able to decrease IL-8 secretion in presence of TNFα. While EVs from MOM_EV_ and PMOM_EV_ were both able to induce a tolerogenic M2-like phenotype in THP-1 macrophages, a significant decrease in the transcript levels of IL-10 and RNA sensing genes was observed with PMOM_EV_. Together, our data indicates that pasteurization of MOM impacts the integrity and functionality of MOM_EV_, decreasing its EVs-mediated immunomodulatory activity. This data provides biomarkers that may be utilized during the optimization of milk processing to preserve its bioactivity.

## Introduction

Human milk (herein referred to as Mother’s own milk, MOM) is a complex source of nutrition and metabolic-endocrine regulators that program the signaling system of infants in a very personalized manner. The bioactive components responsible for these benefits include a wide variety of cytokines, hormones, immunoglobulins, miRNAs, and bacterial cells that are transmitted from mother to child^1, 2^. Our group and others have reported that around 200 different bacterial species have been identified in MOM, suggesting that these may not be simply contaminants but integral components that play a beneficial role in development^3, 4^. MOM provides important benefits to preterm infants, including immune benefits, nutrition, protection against infections (especially necrotizing enterocolitis), and overall, a decreased risk of mortality^5^.

In the absence of MOM, the American Academy of Pediatrics (AAP) recommends using pasteurized donor human milk (DHM) over formula in very preterm infants^6^. Current practice in the Neonatal Intensive Care Unit (NICU) at Shands Children’s Hospital, (University of Florida), is to provide DHM to premature infants of gestational age less than 30 weeks. DHM is obtained from pooled donors and pasteurized, rendering the milk nearly devoid of personalized live commensal bacteria^7^. Furthermore, pasteurization of DHM has also been reported to significantly decrease the levels of sIgA, alkaline phosphatase, bile salt stimulated lipase, lactoferrin and leptin^8, 9^. Nonetheless, the role of the pasteurization process on the diversity of microRNA (miRNAs) has not been evaluated until now. miRNAs are small, non-coding RNAs (around 22-nucleotides in length) that have been found in bacteria, animals, and some viruses. More than 3000 recognized or new miRNAs have been identified in human milk^10, 11^, most of which have been implicated in immune functions.miRNAs are secreted within milk exosomes or extracellular vesicles (EV). EVs have an endocytic origin and are around 30–150 nm (diameter) in size. They are surrounded by a lipid bilayer membrane and contain a rich cargo composed of proteins, DNA, RNA, peptides, and lipid-derivatives^12, 13^. These nanostructures have been isolated from many biological fluids including MOM. Among the rich cargo, miRNAs are considered an outstanding feature due to their role as modulators of a wide variety of processes^14, 15^. It has been reported that miRNAs can regulate more than 30% of human genes^16^. Thus, the transfer of exosomes and their miRNA cargo from mother to child contributes to the benefits provided by breastfeeding. miRNAs contained in MOM EVs have high stability at low pH, suggesting that these regulators could survive the gastric transit, reaching the intestine where they could modulate the host’s immune system^17^. Recently, it was shown that pasteurized or milk subjected to high pressure milk processing introduced shifts in the abundancy of several miRNA^18^. This article overcomes a limitation identified in previous reports where the effect of pasteurization on the functionality of miRNAs contained in the exosomes was not evaluated. The most abundant miRNAs found in MOM target several processes important for infant development (reviewed in^19^). Some of those are: miR-182-5P, miR-148a-3p, let-7f-5p and miR-22-3p, targeting genes involved in lipid metabolism; miR26a and miR181b involved in glucose metabolism; miR21 and miR-200 family participating in tight junction stability in the gut; miR-29b and the let-7 family affecting neurogenesis; and the miR29 and miR-148a-3p implicated in epigenetic regulation. Furthermore, miRNAs miR-181a, miR223, miR146b-5p, and miR155 play important roles in the development of both innate and acquired immune systems.

The preservation of these bioactive components in MOM is highly important in preterm infants, where MOM feedings reduce the incidence and severity of infections, especially necrotizing enterocolitis (NEC) and late onset sepsis (LOS)^20, 21^. As indicated above MOM feedings have shown superior epidemiological performance when compared to DHM and formula. In this report, we have evaluated the effect of the pasteurization process of MOM on the miRNA content of MOM EVs and their impact on innate immune response in human cell lines. We found that pasteurization of MOM impacts the integrity and the miRNA diversity in the EV cargo. Furthermore, biomarkers of host responses that may be followed during the milk processing to evaluate its bioactivity were identified.

## Results

### Pasteurization does not affect the EVs yield of MOM

**Figure 1:**
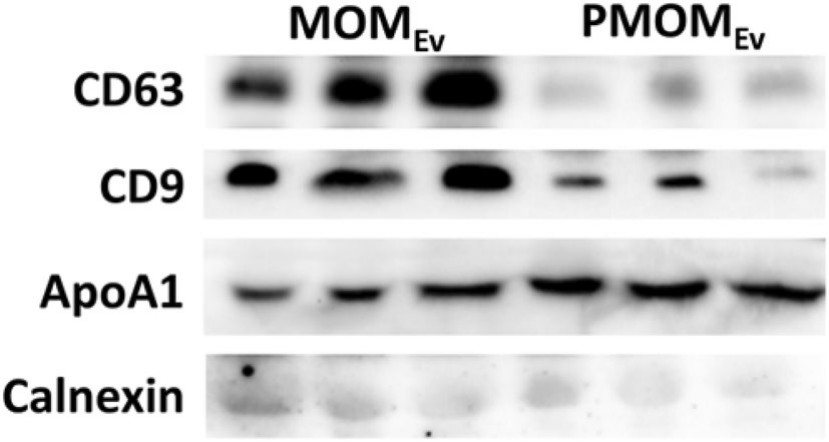
Pasteurization affects the detection of endocytic EV markers. Western blot analyses were performed on total protein extractions of MOM_EV_ and PMOM_EV_ enriched fractions using anti-CD63, anti-CD9, as well as anti-Calnexin as a negative control and anti-ApoA1 as a lipoprotein marker.

To evaluate the role of pasteurization on the stability of miRNAs in human milk, we first developed PMOM (as a proxy for DHM) in which the components are identical to MOM, except that it has been subjected to pasteurization (see “Material and methods” for details). Next, the EVs enriched fractions of MOM and PMOM were assessed for yield and size distribution. A comparable yield of EVs was observed between MOM_EV_ and PMOM_EV_ with an average concentration of 1.6 × 10^10^ ± 9.6 × 10^8^ and 1.7 × 10^10^ ± 1 × 10^9^ EVs per mL of milk, respectively (Suppl. Fig. 1). MOM_EV_ and PMOM_EV_ also showed a similar size distribution (200 nm in average diameter), and negative zeta potential (MOM_EV_ = − 13.4 mV, PMOM_EV_ = − 16.64 mV, respectively). However, immunoblots using the exosome common markers CD63 and CD9 showed that only CD9 was detected in MOM_EV_ and PMOM_EV._ On the contrary, CD63 was observed in MOM_EV_ samples but was absent in PMOM_EV_, indicating damage or degradation during the pasteurization process. The ER marker Calnexin was not detected in either EVs samples, however the apolipoprotein A1 was detectable in both MOM_EV_ and PMOM_EV_, indicating that lipoproteins were co-purified with human milk EVs (Fig. 1).

### MOM_EV_ and PMOM_EV_ are enriched with different miRNA cargo

**Figure 2:**
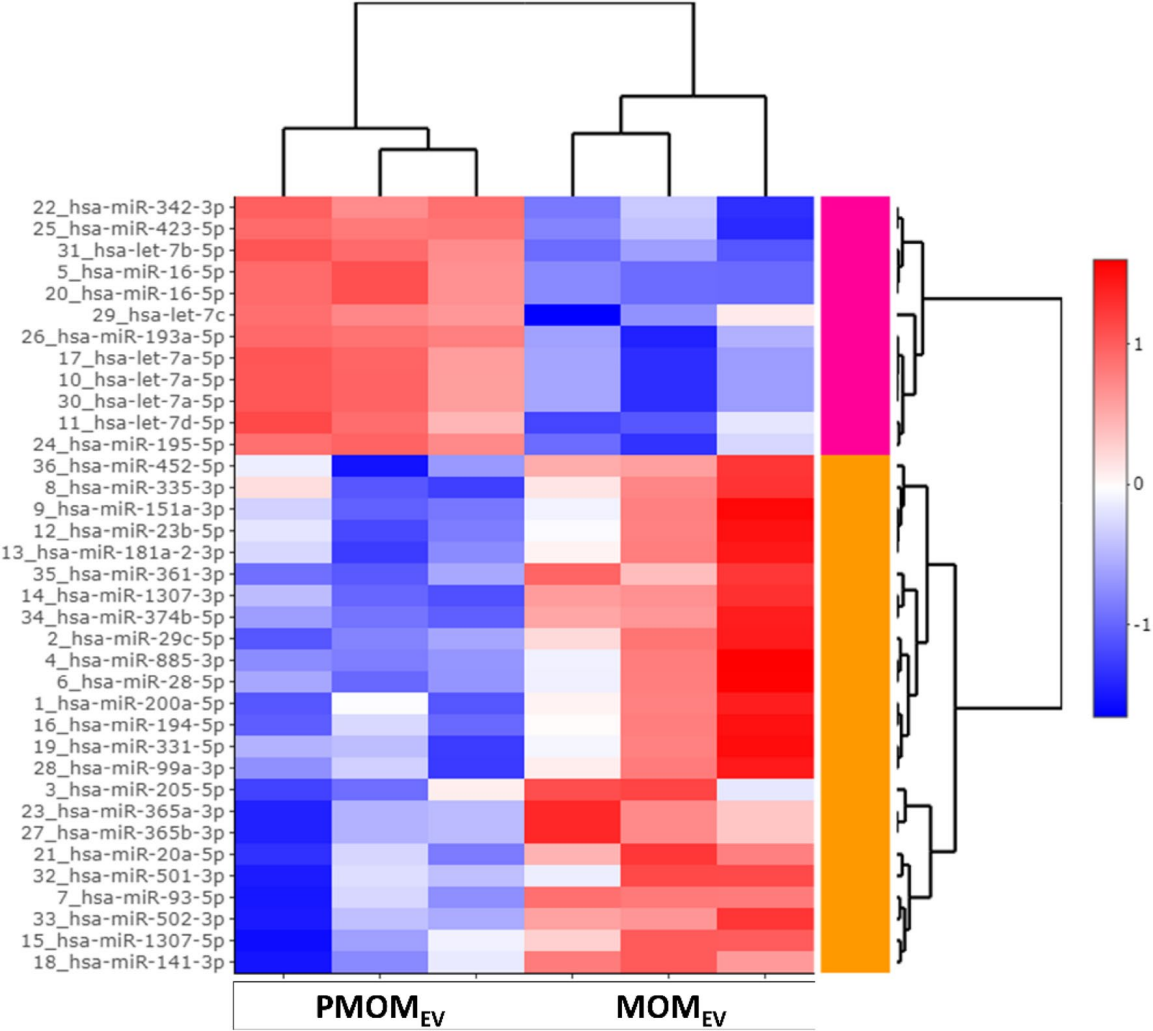
Heatmap summarizing the miRNA sequencing analysis. The distribution of the 33 miRNAs statistically significant enriched in MOM_EV_ and PMOM_EV_ are shown.

**Figure 3:**
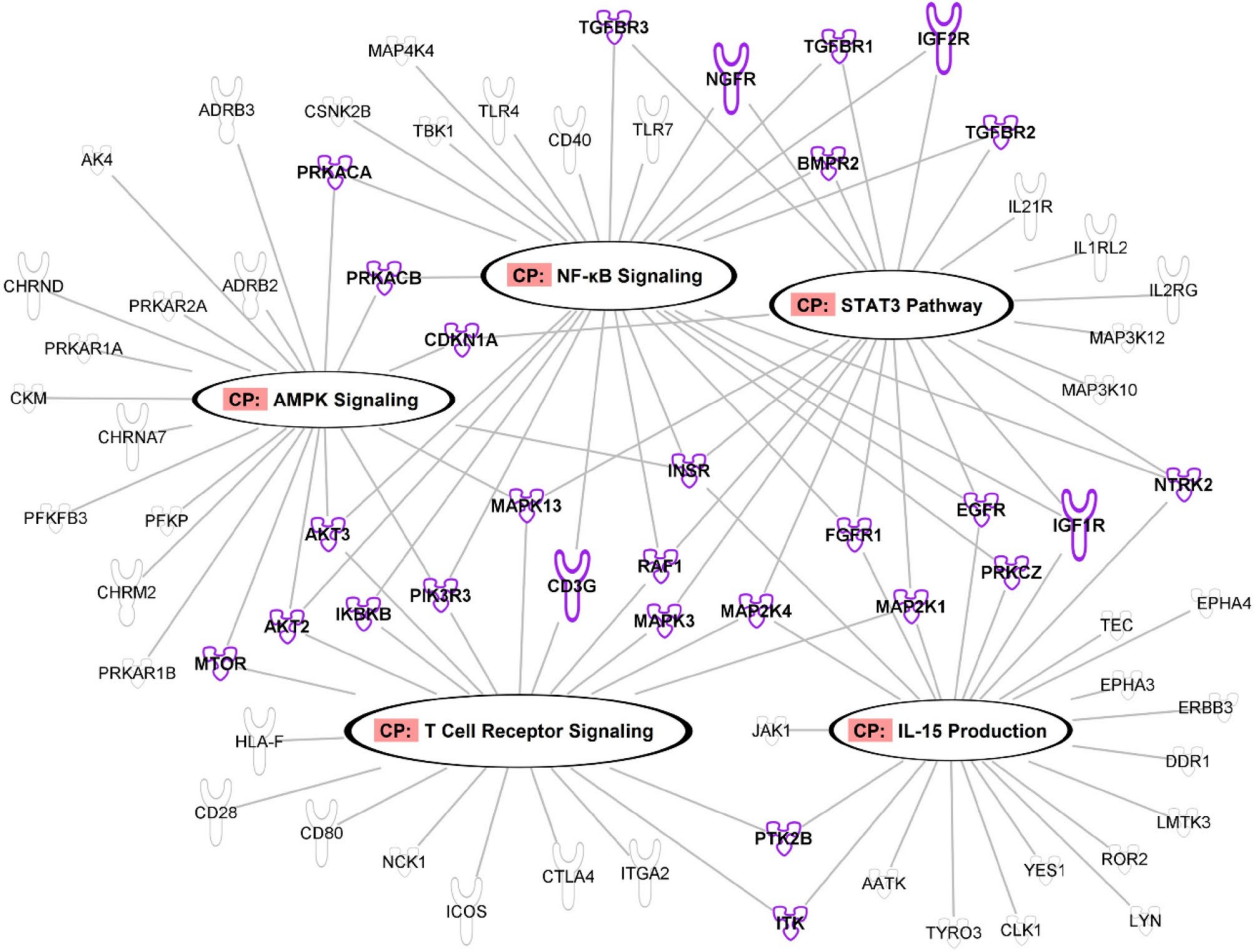
Summary of the miRNA target filter Analysis using IPA Software. The mRNA predicted targets of the miRNA enriched in MOM_EV_ and PMOM_EV_ were filtered using a high confidence level. The pathways involved were filtered by cell proliferation and immune modulators. The network was built with the different effectors targeted by the 33-miRNA enriched in MOM_EV_ and PMOM_EV_. Highlighted in black ovals are the 5 different pathways affected, in bold font and highlighted in purple are the 28 effectors shared by these different pathways. The pathways and targets were generated using QIAGEN Ingenuity Target Explorer (QIAGEN, Inc., https://targetexplorer.ingenuity.com/).

Global miRNA RNAseq was performed on MOM_EV_ and PMOM_EV_ enriched samples. A total of 166 miRNA were identified with 33 miRNAs of those being differentially enriched. It was found that 9 miRNAs were enriched in PMOM_EV_ while 24 were enriched in MOM_EV_ (Fig. 2, Suppl. Table 1). To further understand the potential impact of the differences observed between MOM_EV_ and PMOM_EV_, we utilized the microRNA target filter tool using the Ingenuity Pathway Analysis software (IPA) (Table 1). Of the differentially enriched mRNAs, 25 were predicted to target 201 messenger RNAs. About fifty percent of those genes (92 mRNAs), are involved in five different pathways: AMPK signaling, NF-κB signaling, STAT3 pathway, T Cell Receptor signaling, and IL-15 production (Fig. 3). The analyses of those five pathways showed that they share 28 genes that may be affected by the miRNAs differentially enriched in MOM_EV_ and PMOM_EV_.

**Table 1:** Statistically significant miRNA enriched differentially between MOM and PMOM EVs.

miRNA	logFC	Adj. *P* value
hsa-let-7b-5p	4.8	2.85E−06
hsa-let-7a-5p	3.7	4.54E−04
hsa-let-7a-5p	3.7	4.54E−04
hsa-miR-342-3p	3.3	4.54E−04
hsa-miR-16-5p	3.3	6.30E−04
hsa-let-7c	2.9	6.81E−03
hsa-let-7d-5p	2.9	6.57E−03
hsa-miR-193a-5p	2.9	3.84E−03
hsa-miR-195-5p	2.5	9.31E−03
hsa-miR-423-5p	2.1	9.45E−03
hsa-miR-20a-5p	− 2.5	9.31E−03
hsa-miR-93-5p	− 2.6	9.31E−03
hsa-miR-885-3p	− 2.9	9.31E−03
hsa-miR-28-5p	− 2.9	9.31E−03
hsa-miR-151a-3p	− 3.0	9.31E−03
hsa-miR-331-5p	− 3.0	9.45E−03
hsa-miR-141-3p	− 3.1	2.63E−03
hsa-miR-361-3p	− 3.2	2.63E−03
hsa-miR-194-5p	− 3.3	7.54E−03
hsa-miR-23b-5p	− 3.3	6.83E−03
hsa-miR-1307-5p	− 3.4	5.41E−03
hsa-miR-501-3p	− 3.4	8.27E−03
hsa-miR-374b-5p	− 3.4	2.41E−03
hsa-miR-200a-5p	− 3.5	7.54E−03
hsa-miR-365a-3p	− 3.6	4.54E−04
hsa-miR-365b-3p	− 3.6	4.54E−04
hsa-miR-99a-3p	− 3.7	3.84E−03
hsa-miR-205-5p	− 3.8	3.03E−04
hsa-miR-452-5p	− 3.8	2.63E−03
hsa-miR-181a-2-3p	− 3.8	2.98E−03
hsa-miR-502-3p	− 3.9	1.86E−03
hsa-miR-335-3p	− 4.4	2.51E−03
hsa-miR-1307-3p	− 4.4	4.54E−04
hsa-miR-29c-5p	− 4.5	8.79E−04

Positive log fold change (logFC) shows enriched miRNA in PMOM_EV_, while negative fold changes show enriched miRNA in MOM_EV_.

**Figure 4:**
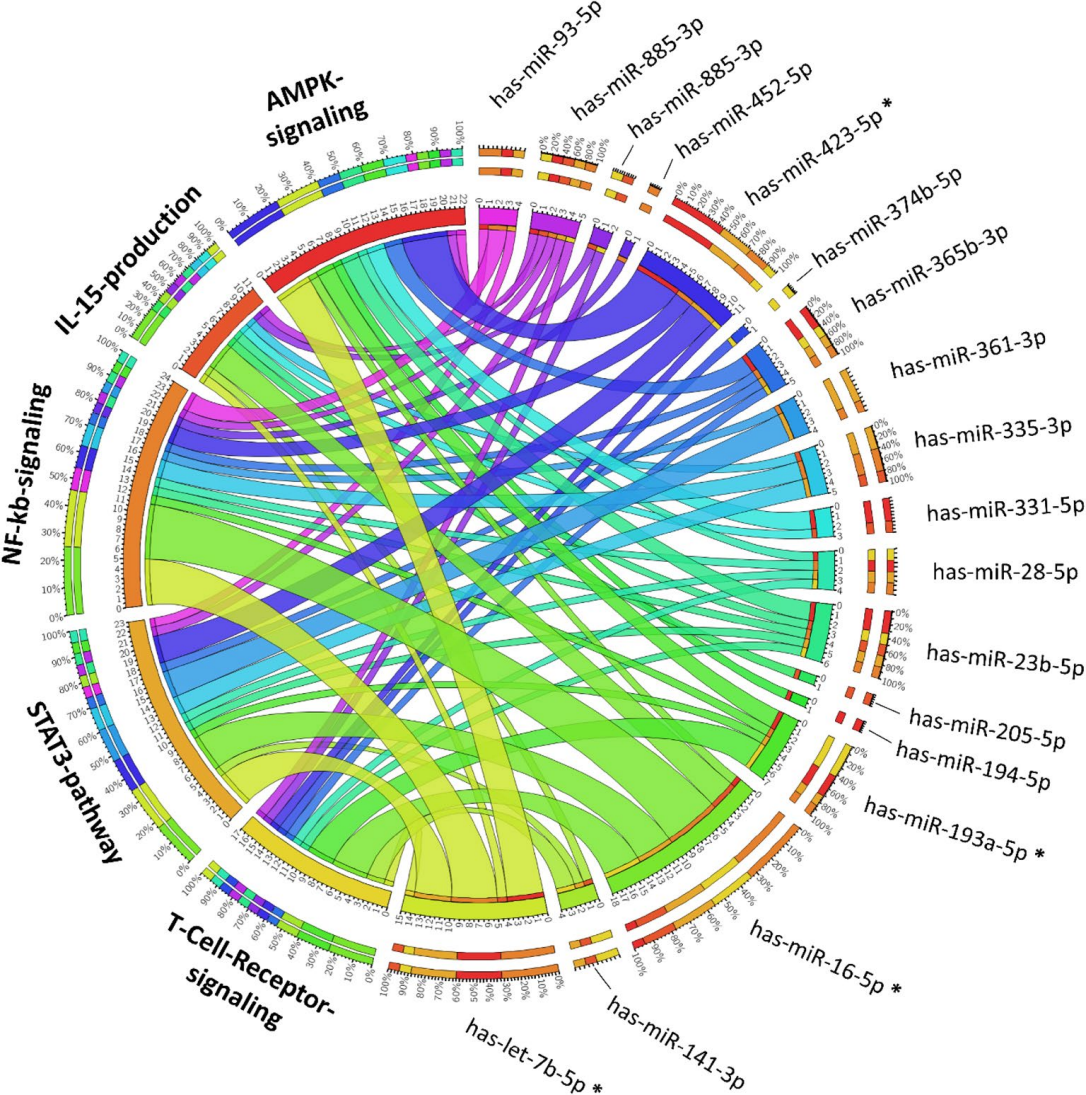
Circos Plot representation to visualize miRNA-gene and inference of the biological impact in the different pathways. With “*” are denoted the miRNA enriched in PMOM_EV_.

Specifically, the miRNAs enriched in MOM_EV_ target 41 mRNA while the miRNAs enriched in PMOM_EV_ target 51 genes (Suppl. Table 2). To integrate and visualize the IPA results obtained, we used Circos plot to combine in a single plot the potential pathways affected by miRNA from both MOM_EV_ and PMOM_EV_ (Fig. 4). As shown in the Circos plot, only has-let-7b-5p, has-miR-16-5p, has-miR-193a-5p, and has-miR-423-5p identified in PMOM_EV_ are predicted to have an impact on these pathways while 14 miRNAs from MOM_EV_ are predicted to impact the mRNA involved in the same pathways (Figs. 3 and 4, Table 1 and Suppl. Table 2). Based on these predictions, miRNAs enriched in PMOM_EV_ may have a greater influence in the regulation of the transcription factor NF-κB signaling pathway miRNAs enriched in MOM_EV_ may have a greater influence in the regulation of IL-15 signaling pathway (Fig. 4). However, both pathways are essential in the development and modulation of multiple aspects of the innate and adaptive immune system^22, 23^. These results suggest that miRNA differentially enriched in MOM_EV_ and PMOM_EV_ may affect the immune system through different but overlapping mechanisms.

### EVs from MOM and PMOM stimulate IL-8 secretion in Caco2 cells

**Figure 5:**
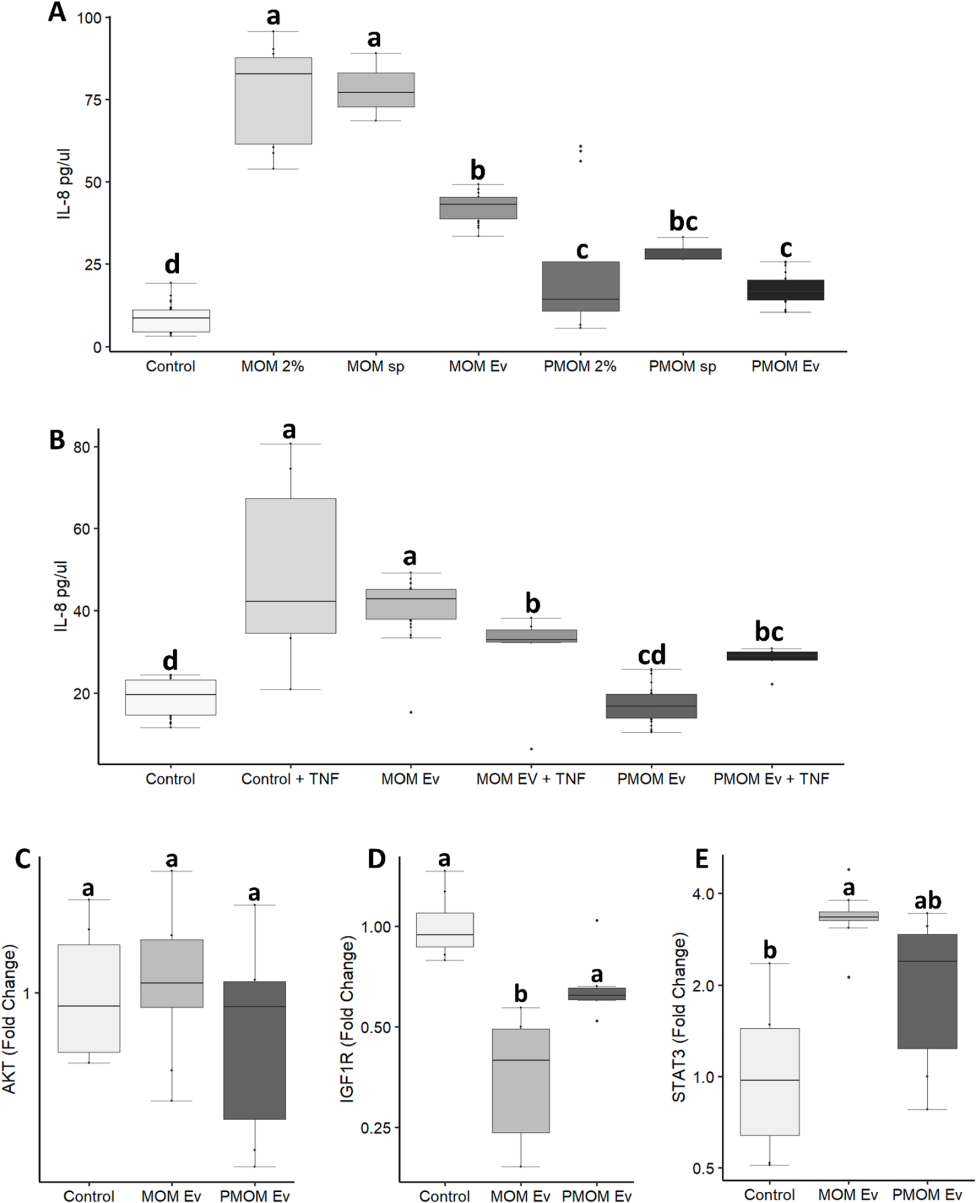
Effect of human milk EVs on stimulation of the innate immune response in Caco-2 epithelial cell line. (**A**–**B**) ELISA quantification of IL-8 in Caco2 epithelial cells after 6 h of treatment with **A**) different milk fractions or **B**) with human milk enriched EV fractions in presence and absence of TNFα. Untreated Caco2 cells were used as control. (**C**–**E**) Relate expressions of *AKT* (**C**), *IGF1R* (**D**), and *STAT3* (**E**) are shown as fold change relative to the vehicle control. The experiments were performed with biological and technical triplicates. One way ANOVA were performed, different letters indicate statistical significance at *p* < 0.05.

Based on the previous predictions, the impact of enriched MOM_EV_ and PMOM_EV_ fractions was evaluated on the nuclear factor kappa B (NF-κB) pathway by following the induction of IL-8 expression^24^. As controls, whole MOM and PMOM as well as exosome free supernatants were tested. A total of 24 fresh MOM samples were pooled into 3 groups and their ability to stimulate IL-8 secretion in Caco2 cells was evaluated. Differential fractionation was performed to evaluate whole milk, EV free supernatant and EV enriched fractions. It was found that while all the treatments induced secretion of IL-8 in Caco2 cells compared to the control, significantly higher secretion levels of IL-8 were observed in Caco2 cells treated with MOM 2%, supernatant and MOM_EV_ when compared to their corresponding PMOM fractions (Fig. 5A). The results obtained with the supernatants were expected as the milk contains a variety of cytokines that may nonspecifically stimulate IL8 secretion.

The effect of the human milk EVs was further investigated in a proinflammatory environment by adding TNFα to Caco2 cells in presence or absence of EVs. It was found that MOM_EV_ had similar secretion levels of IL-8 as the TNFα control, while the addition of TNFα to Caco2 in presence of MOM_EV_ was able to decrease IL-8 secretion. In contrast, PMOM_EV_ showed trends (albeit not significant *p* value = 0.055) to increase IL-8 in presence of TNFα in Caco2 cells, (Fig. 5B). TNFα is a positive regulator of IL-8 gene expression through NF-κB, which is essential for IL-8 gene transcription^24^. These results are in agreement with predicted regulatory effect of PMOM_EV_ miRNAs on the NF-κB signaling pathway (Fig. 4).

Another predicted signaling pathway affected by the miRNAs enriched in both MOM_EV_ and PMOM_EV_ is STAT3 Pathway (Figs. 3 and 4). The Signal Transducer and Activator of Transcription (STAT) 3 pathway is involved in survival, cell growth, and immune response^25^. This pathway is rigorously regulated by Janus Kinase (JAK) and Epidermal Growth Factor Receptor (EGFR). We identified EGFR as one of the targets regulated by hsa-miR-16-5p enriched in PMOM_EV_. In order to elucidate and further understand if the EVs from human milk were able to differentially modulate these pathways, we determined the mRNA expression of some key effectors such as *AKT*, *STAT3*, and *IGF-1* receptor, in Caco2 cells treated with MOM_EV_ or PMOM_EV_. A significant decrease in the expression of *IGF*-1 receptor was observed with both EVs treatments while no significant differences were observed in the expression of *AKT* compared to the control (Fig. 5C–D). Additionally, a 3.5-fold increase was observed in the expression of *STAT3* after treating Caco2 cells with MOM_EV_. While the expression of *STAT3* was higher in the cells treated with PMOM_EV,_ no significant differences were observed when compared to the control (Fig. 5E). However, when STAT3 was evaluated by Western blot, no significant differences were observed between the treatments and the control (Supplementary Fig. 2). These results suggest that regulation of *STAT3* transcript could be modulated in a hsa-miR-16-5p through EGFR repression in Caco2 cells treated with PMOM_EV_.

### Differential M2 tolerogenic differentiation of THP-1 human macrophages by MOM_EV_ and PMOM_EV_

**Figure 6:**
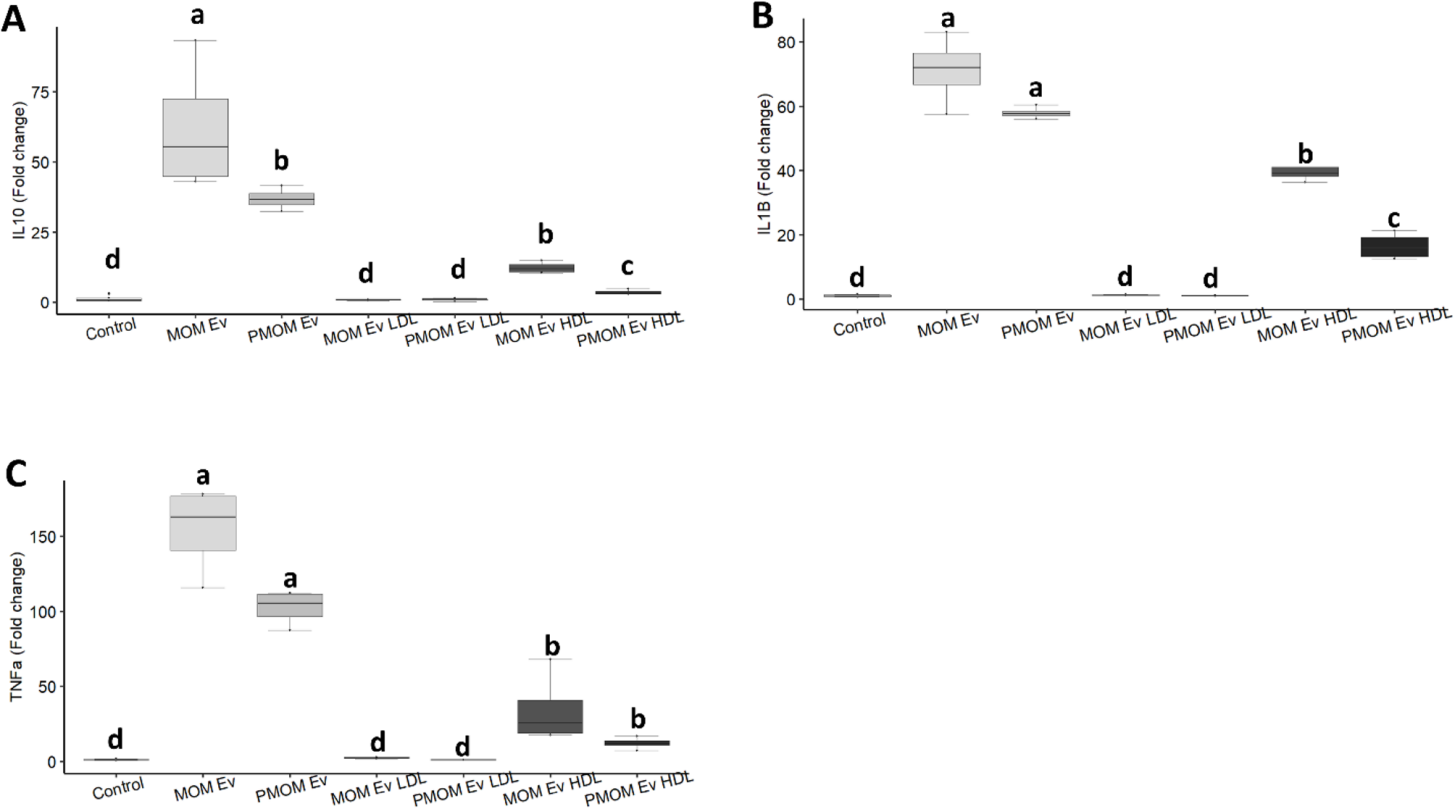
Human milk EVs stimulate a M2-tolerogenic phenotype in THP-1 macrophages. The relative expression of *IL-10* (**A**), *TNFα* (**B**) and *IL1β* (**C**) are shown relative to the vehicle control. The experiments were performed with biological and technical triplicates. ANOVA and mean comparisons were performed, different letters indicate statistical significance for *p* < 0.05.

The potential impact of the enrichment of has-let-7b-5p in PMOM_EV_ was evaluated on the mRNA expression levels of IL10^26^ using the THP-1 PMA-activated macrophage cell line. Macrophages are broadly located in the human body playing a crucial role in regulating immune responses^27^. Once activated, macrophages can differentiate into two sub-types M1-like macrophages capable of proinflammatory responses and M2-like macrophages capable of tolerogenic responses^28^. To this end, THP-1 monocytes induced with PMA were treated with MOM_EV_ and PMOM_EV_ at 10^10^ particles/well for 6 h. The mRNA levels of IL-10, TNFα and IL-1β were quantified as signature markers of tolerogenic THP-1 (M2) macrophages. It was found that both MOM_EV_ and PMOM_EV_ induced a M2 phenotype when compared to the vehicle control. Nonetheless, THP-1 macrophages treated with MOM_EV_ had significantly higher levels of IL-10 expression when compared to PMOM_EV_ (Fig. 6A–C). These findings are in agreement with the higher abundance of has-let-7b-5p in PMOM_EV_.

As lipoproteins were observed with MOM_EV_ and PMOM_EV_, their contribution to the M2b phenotype observed was investigated. LDL (Low-density lipoprotein) and HDL (High-density lipoprotein) lipoproteins from MOM_EV_ and PMOM_EV_ enriched fractions were extracted and tested at equivalent concentrations to those found in the EVs. The addition of MOM_HDL_ or PMOM_HDL_ significantly induced expression of TNF-α, IL1β and IL-10 when compared to the vehicle control (Fig. 6A–C). However, the induction levels were significantly lower than those compared to the MOM_EV_ or PMOM_EV_. It was found that the addition of MOM_LDL_ or PMOM_LDL_ resulted in a similar response as the vehicle control for all the genes tested. These results indicate that lipoproteins found in the EV enriched fractions partially contribute to the tolerogenic M2b phenotype.

**Figure 7:**
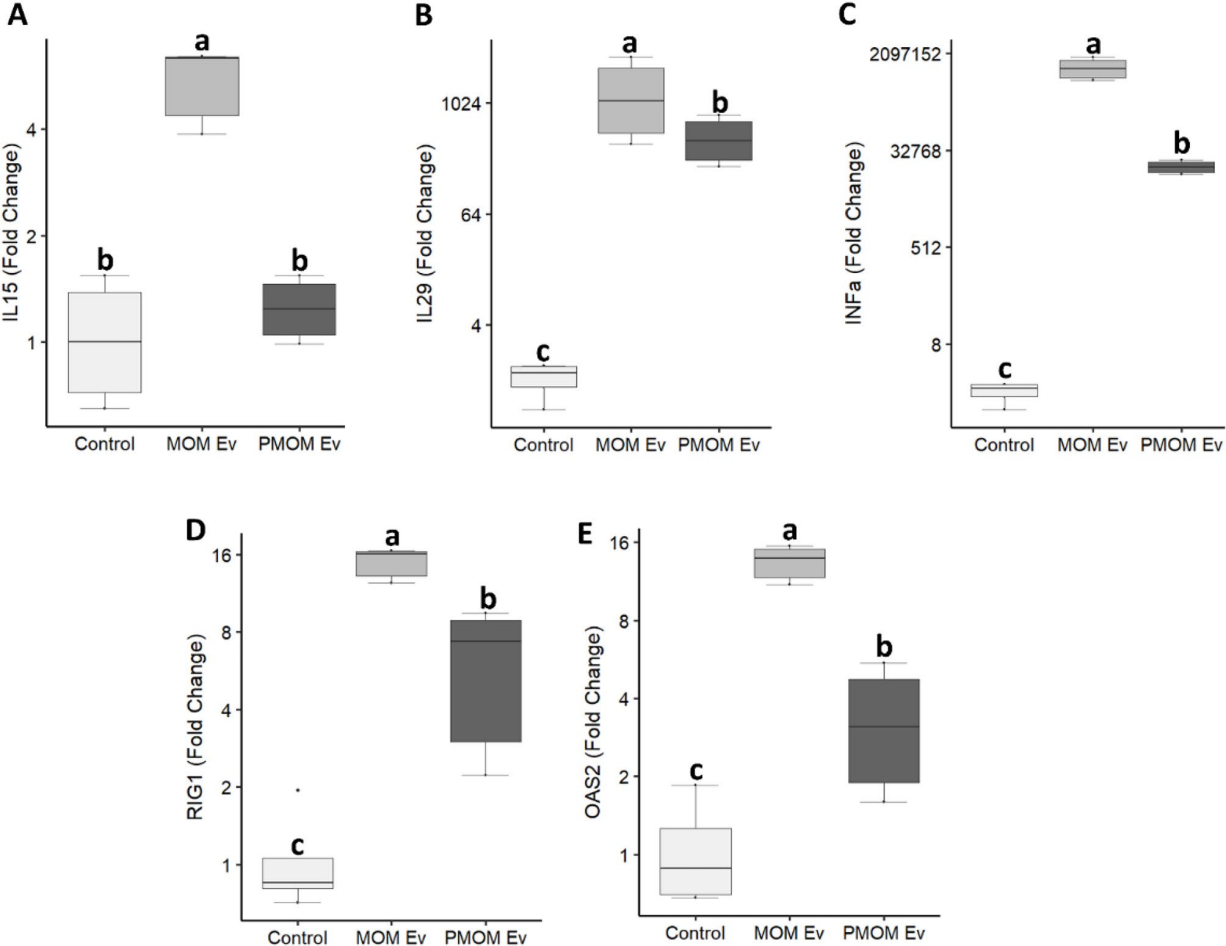
Human milk EVs stimulate gene expression of RNA sensing signaling in THP-1 macrophages. The relative expressions of *IL-15* (**A**), *IL29* (**B**), *INFα* (**C**), *RIG1* (**D**) and *OAS2* (**E**) are shown as fold change relative to the vehicle control. The experiments were performed with biological and technical triplicates. Single way ANOVA and mean comparisons were performed, different letters indicate statistical significance at *p* < 0.05.

Next, we investigated the impact of milk EVs on the IL-15 production pathway (Figs. 3 and 4). IL-15 is a master regulator playing a crucial role in inflammatory and protective immune response^29, 30^. IL-15 has pivotal role as a signal molecule shared by several pathways like JAK/STAT pathway, Ras/MEK/MAPK pathway, PI3K/Akt/mTOR pathway and NF-κB pathway^31^. Interestingly, significant expression levels of IL-15 mRNA were observed in THP-1 macrophages treated with MOM_EV_ but not with PMOM_EV_ (Fig. 7 A) or with purified lipoproteins (Suppl. Fig. 4). These results are in agreement with the predicted regulatory role of has-let-7b-5p and has-miR-16-5p, enriched in PMOM_EV,_ on the NF-κB signaling pathway.

IL-15 in humans is further regulated by IFN-γ (also known as IL-29) which is part of the IL-10-family of cytokines. This type III interferon exhibits similar type I interferon-like RNA sensing properties resulting in activation of the JAK/STAT pathway^32, 33^. Additional inducers of interferon are the sensing of RNA by retinoic acid-inducible gene I (RIG-I)^34^ and 2’-5’-oligoadenylate synthetase 2 (OAS2). Therefore, to further evaluate the impact of differential miRNA cargo in human milk EVs, in RNA sensing pathways and interferons, the mRNA levels of IL29, OAS2, RIG-I and INF-α were determined. Although both treatments, MOM_EV_ and PMOM_EV_, induced higher expression of these genes when compared to the control, significant higher levels of OAS2, INF-α and IL29 were observed in THP-1 macrophages treated with MOM_EV_ compared to PMOM_EV_ for *p* < 0.05 (Fig. 7). Altogether, these results indicate that the miRNA cargo of human milk EVs could be compromised after the pasteurization process decreasing or changing the regulatory effect exerted by human milk miRNA.

### MOM_EV_ differentially affects AMP, IKBα and STAT3 signaling pathways

**Figure 8:**
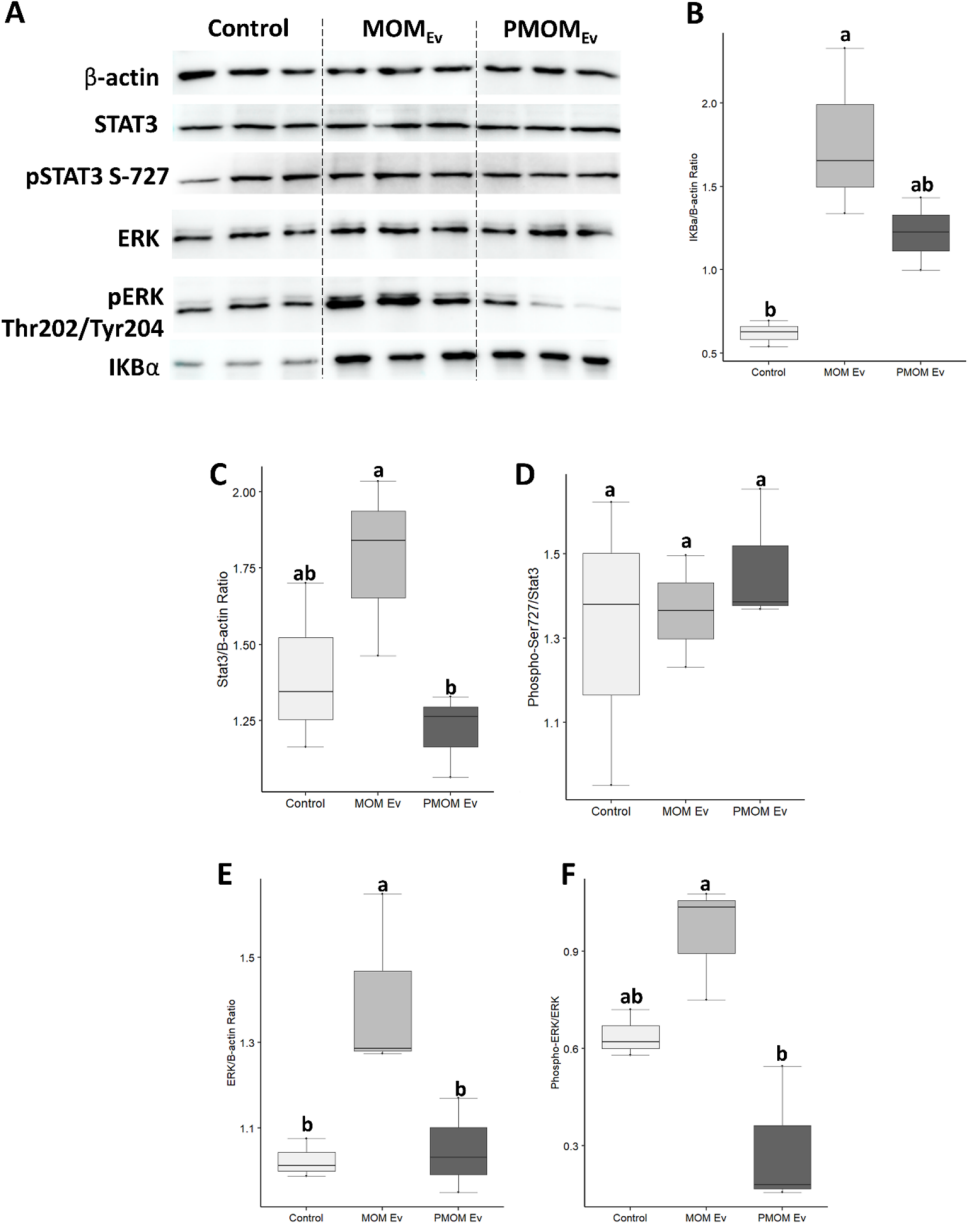
Effect of MOM_EV_ and PMOM_EV_ on AMP, IKBα and STAT3 signaling pathways. (**A**) Summary panel of the Western blots performed after incubation of THP-1 macrophages in presence or absence of MOM_EV_ and PMOM_EV_. The results of biological triplicates are shown. (**B**–**F**) Quantification of the results obtained with (**B**) total IkBα, (**C**) total STAT3, (**D**) STAT3 phosphorylation S-727, (**E**) total ERK, and (**F**) ERK phosphorylation Thr202/Tyr204. β-actin was used as a loading control. Quantification of band intensity was performed with ImageJ. One way ANOVA were performed, different letters indicate statistical significance at *p* < 0.05.

The impact of MOM_EV_ and PMOM_EV_ on AMPK signaling, NF-κB signaling (by following the protein basal level and activation of the IkB kinase), and STAT3 pathways, was confirmed by evaluating the protein levels in THP-1 macrophages. It was found that the basal level abundancy of ERK and IkBα were significantly higher in MOM_EV_ when compared to the control (Fig. 8). STAT3 showed a similar trend albeit not statistically significant. PMOM_EV_ showed significantly lower concentrations of STAT3 and ERK when compared to MOM_EV_ while IkBα showed a similar trend albeit not statistically significant. The quantification of the activating phosphorylation’s in ERK (P-Thr202/Tyr204 ERK/ERK) showed a significant decrease in PMOM_EV_ when compared to MOM_EV_. No significant differences were observed for P-S727 STAT3/STAT3 between MOM_EV,_ PMOM_EV_ and the vehicle control (Fig. 8). These results are in agreement with the stronger stimulation of innate immune responses observed with MOM_EV_.

## Discussion

Many mothers that deliver preterm do not produce sufficient volumes of MOM^35^, and as a result, DHM is provided for these infants due to the increased benefits over formula^6^. However, the pasteurization process of DHM significantly reduces the availability of bioactive molecules as well as personalized live commensal bacteria^9^. In this report, we evaluated the impact of pasteurization on human milk on EVs integrity and miRNA cargo. We found that while similar yields and charge of EVs were obtained for MOM and PMOM EVs, a significant decrease in the protein marker CD63 was observed in PMOM_EV_, while CD9 was detectable in both EVs samples. Other ER markers such as calnexin were not detected on either sample while the Apolipoprotein A1 was present in both MOM_EV_ and PMOM_EV_. Lipoproteins and EVs are both in the submicron scale and overlap in size distributions which translates frequently in co-purification of EVs and lipoproteins^36, 37^. The presence of lipoproteins could explain the comparable yield and size between MOM_EV_ and PMOM_EV_ samples. Recent reports in bovine milk-derived EVs evaluated the impact of several industrial processes on EV integrity. Similar to our findings, pasteurization and homogenization significantly affected the levels of CD63 while CD9 was heat stable^38^. Other studies have shown that the stability of exosomes is highly affected by low temperatures as well^39^.

Our RNAseq approach also identified significant changes to the miRNA diversity in EVs of MOM upon pasteurization. These results suggest that the decrease in protein stability of the EVs was translated into changes in the miRNA cargo in human milk. The stability and shifts in the miRNA cargo upon heat treatments procedures such as pasteurization and ultra-heat treated (UHT) milk has been studied in human and bovine milk with conflicting results^38^. Smyczynska et al.^18^ observed more severe effects of pasteurization of DHM, using Holder Pasteurization (HoP) than with High-Pressure Processing (HPP). A 302-fold decrease in the yield of exosomes was observed with (HoP), with a complete loss of RNA fragments of length typical for miRNA and piRNA. High pressure processing DHM showed less detrimental effect than pasteurization in human milk^18^. However, the impact of these results on host responses were not evaluated. Some studies have reported a significant effect in the abundance and stability of miRNA in bovine milk^38, 40^, while others have shown that pasteurization does not have a significant effect^41, 42^. These conflicting results may be explained by the differences in methods used for their analyses.

We determined the differential enrichment of 33 miRNA between MOM_EV_ and PMOM_EV_. Moreover, the main five pathways predicted to be impacted by the differentially enriched miRNA share multiple genes resulting in overlapping effects on immune stimulation and cell proliferation. To evaluate the impact of the potential loss in integrity or decreased abundancy of specific miRNA, we used intestinal epithelial and macrophage cell lines to measure key gene markers. The miRNA enriched in MOM_EV_ and PMOM_EV_ target 25 genes involved in NF-κB signaling pathway. Of those, only six genes are shared among MOM_EV_ and PMOM_EV_ (CD40, CXCR5, PIK3R3, TGFBR1, TGFBR2 and TLR4). We hypothesized that the differential enrichment of miRNA after pasteurization would affect signaling through the NF-κB pathway. To this end, we followed the expression of IL-8 in human epithelial cells^24^. Stimulation of IL-8 secretion was observed with all the fractions of human milk tested (human milk, supernatant free of EVs and EVs), however, significant difference in the stimulation of IL-8 secretion was observed when fractions from PMOM were compared with their correspondent MOM fractions. Similar findings have been observed in the stimulation of IL-8 secretion after heat treatment of bovine and goat milk EVs^43, 44^. Likewise, a reduction in the expression of IL-6 was observed in a mice model of necrotizing enterocolitis (NEC) when comparing raw and pasteurized human milk EVs^45^.

Another predicted pathway showing overlapping effector genes was the MAPKs and PI3K/AKT. To further investigate the effects of MOM_EV_ and PMOM_EV_ in Caco2 cells, we evaluated the expression levels of 3 key genes involved in these pathways. Lower expression levels of IGF1R were observed with both MOM_EV_ and PMOM_EV_ treatment in Caco2 cells. However, only MOM_EV_ showed statistically significant differences. IGF1R activation mediates signaling cascades through MAPKs and PI3K/AKT^46, 47^ impacting many cellular responses including cell proliferation, differentiation, and survival^46, 47^. We found that the posttranscriptional regulators of IGF1R, hsa-miR-16-5p and hsa-let-7b-5p, were enriched in PMOM_EV_, consistently with the lower levels in IGF1R mRNA observed. However, the decrease in expression did not reach statistical significance. The indirect compensatory effect of increased expression levels of STAT3 observed could explain those observations.

The beneficial effects of human milk on immune stimulation have been largely described^1, 2^, however little is known about the immune stimulatory properties of human milk EVs. In bovine milk, the lactation-related differential expression of miRNAs suggests that the miRNA produced in the mammary glands may have a specific function^48^. Since many of the enriched miRNA in human milk EVs identified in this work are predicted to impact immune modulatory pathways, we evaluated the impact of MOM_EV_ and PMOM_EV_ on the stimulation of a M2-like tolerogenic phenotype in macrophages. Interestingly, a stronger stimulation of IL-10 (as gene expression levels) was observed in macrophages treated with MOM_EV_ compared to PMOM_EV_. These results concur with the predicted activation of the canonical NF-κB signaling pathway through has-let-7b-5p and has-miR-16-5p enrichment quantified in PMOM_EV_. While the tolerogenic M2-like polarization of macrophages observed in this work has been described earlier for other miRNAs^17, 49^, macrophage differentiation and responses could be tissue- and species-dependent. In example, inflammatory M1-like macrophage differentiation, characterized by high levels of IL-6, TNFα, IL-12/23 and decreased IL-10, was observed with bovine milk-derived EVs (BEVs) using a mice model for agricultural dust exposure^50^. In contrast, BEVs administration in two mouse models for arthritis reduced serum levels of MCP-1 and IL-6 correlated with delays in the onset of arthritis and diminished cartilage pathology and bone marrow inflammation^51^.

A remarkable finding was the induction of IL15 in macrophages treated with MOM_EV_. This cytokine has protective roles towards viral and bacterial infections^52, 53^. IL15 has been also successfully used as adjuvant in many antiviral vaccines^23^. The stimulation of IL15 positively correlated with higher expression levels of the interferons IL29, INFα, and the RNA sensing genes RIG1 and OSA2 in MOM_EV_ when compared to PMOM_EV_. The potential role of milk miRNA in viral interference was explored through in silico methods in the context of the SARS-CoV-2 pandemic. It was found that some of the abundant miRNA in milk (miR29a, miR21 and miR181), can interfere with replication of a wide variety of viruses such as HIV, enterovirus 71 and influenza. However, scarce information is available on the mechanisms that mediate these cross-kingdom interactions^11, 19^. Together, these results suggest that human milk EVs may play a significant role in stimulation of RNA sensing pathways to trigger an antiviral response that may be diminished by the pasteurization process.

There is substantial evidence that milk exosomes can be absorbed and delivered to peripheral tissues where they can exert their regulatory effect (For review see Zempleni et al.)^12^. Therefore, the differences observed in the bioactivity of MOM after pasteurization raise concerns regarding the potential impacts of these processes on health outcomes. While the benefits of MOM have been widely reported, the benefits provided by specific bioactive components such as EVs and their cargo in human milk is unknown. It has been shown that physiological concentrations of bovine milk miRNA can affect gene expression in vivo and in cell cultures (Peripheral Blood Mononuclear Cells, HEK-293 Kidney)^54^. These reports suggest that a decrease in MOM EVs concentration or in their integrity affecting their cargo bioactivity can potentially have an impact on the health of infants. The most significant difference observed in this work was a decrease in the stimulation of IL15 as well as RNA sensing genes by PMOM_EV_. The reduced stimulation observed in innate immune responses maybe translated into a decreased response to viral infections. These findings, combined with the reported decreases in numerous proteins (lactoferrin, lysozyme)^55^, immunoglobulin, and cytokines (like IL-7)^56^ reported by others^9, 57^, may significantly impact the overall immune bust usually provided by MOM.

The results presented here highlight the need to optimize processes in human milk banks in order to preserve the potential bioactivity of all components in human milk while maximizing its biosafety. There is no direct substitute for the nutritional and immune benefits that MOM provides, but its proven benefits highlight the need for attempting to replicate these factors in alternative feedings as closely as possible. In this work, we identified mechanistic effectors that may be utilized as biomarkers for process optimization. The decreased immunomodulatory activity of pasteurized milk and its derived EVs observed needs to be addressed in future studies, in order to establish better processing strategies and to provide our VLBW infants with the best and personalized nutrition possible.

## Material and methods

### Milk collection and EV enrichment

For the RNAseq extractions, samples were collected using a sterile Symphony® double breast pump kit at a single expression session with an electric hospital Symphony® breast pump (Medela, McHenry, IL). The protocols used in this study for sample collection were reviewed and approved through the University of Florida Institutional Review Board (IRB RB201400527), a written informed consent was obtained from each donor mother. All methods here were performed in accordance with the relevant guidelines and regulations of the University of Florida Institutional Review Board. Beyond standard hand washing and pumping per NICU protocol, no breast hygiene preparation was performed. The sample was assigned a de-identified subject number, then immediately transported on ice to the microbiology lab for further processing. For the functional analyses using human cell lines, 24 milk samples we obtained from the NICU. Sets of 8 samples (total of 3 sets) were pooled. For both experiments each pool of milk was divided into two fractions, one fraction was immediately processed (MOM) for EV enrichment (MOM_EV_), while the other half was pasteurized to mimic DHM (P-MOM) to obtain PMOM_EV_, following the HMBANA protocol (HMBANA 2020). Briefly, milk was heated at constant temperature 65 °C for 30 min and cooled down immediately after in an ice bath. After pasteurization milk was further processed for EV enrichment as follows. MOM and PMOM fractions were centrifuged at 2246 g for 15 min at 4 °C to remove the fat, following a 45 min centrifugation at 15900 g to eliminate cell debris. Milk supernatants were further filtered sequentially using nitrocellulose filters of 11 um, 6 um, 2.5 um, 0.45 um and 0.2 um. After filtration, EVs were enriched by ultracentrifugation at 207,888 g for 2 h. The EVs were washed twice with PBS, quantified, and stored in aliquots at − 80 °C.

### EV physical characterization and quantification

Nanoparticle tracking analysis (NTA) using a NanoSight NS300 (Malvern Instruments Ltd, Malvern, UK) was utilized to quantify the EVs as well as to determine its size distribution. Videos were recorded for 60 s (five times), with the camera level at 15, and analyzed with NTA software 4.3 (Malvern instruments Ltd, Malvern, UK). An average yield of 10^12^ exosomes per uL was obtained. Dynamic light scattering was performed to measure the zeta potential of the EV suspensions using a Zetasizer ultra particle analyzer (Malvern Instruments Ltd, Malvern, UK). The samples were diluted 1:1000 with distilled water. The measurements were conducted in biological and technical triplicates at 25 °C.

### RNA extraction, sequencing, and data analysis

RNA extractions were performed from three pools of MOM_EV_ and PMOM_EV_ samples using *mir*Vana™ miRNA Isolation Kit (Invitrogen), with small RNA enrichment from total RNA according to manufacturer’s instructions. Library construction with fragments around 20–75 bp in length and sequencing using Ion Torrent sequencing platform was performed by PrimBio (PrimBio Research Institute LLC, Exton, PA). FastQC was used to filter high quality reads^58^. For the data analysis Kallisto v0.46.1^59^ was used to create an index and map the high-quality raw reads using a reference transcriptome for no coding RNA through EnsDb.Hsapiens.v86 2.99.0 package in RStudio^60, 61^. Differential abundance analysis of miRNA was performed using limma 3.52.4, edgeR 3.38.4 and SVA 3.44.0 packages in RStudio^62–64^. All the graphics were generated using ggplot2 3.3.6 R package^65^.

### Protein extractions and Western Blot

Aliquots normalized to the same particle concentration of MOM_EV_ and PMOM_EV_ were used for protein extraction and quantification using the Pierce™ BCA Protein Assay Kit (Thermo Scientific, Rockford, IL). Briefly, total proteins were extracted from EVs using Radio Immunoprecipitation Assay Buffer (RIPA) containing 150 mM NaCl, 50 mM Tris (pH 8), 1% Triton X-100, and 0.1% sodium dodecyl sulfate (SDS), with Halt™ protease inhibitor cocktail (Thermo Fisher, Waltham, MA, USA). The EVs homogenates were centrifuged at 12,000 g for 10 min, at 4 °C and the protein concentration was measured following the manufacturer instruction.

Lipoproteins HDL (High-Density lipoprotein) and LDL (Low-Density lipoprotein) were purified from human milk EVs suspensions using LDL/VLDL and HDL purification kit (STA-608 ultracentrifugation free) following manufacturer protocol (Cell Biolabs, INC). Lipoproteins were solubilized in equal volume of PBS as EVs suspensions.

Human milk EVs were analyzed for exosome surface proteins by Western blot. Anti-CD63 (ab134045) and anti-CD9 (ab263019) were used as positive control. To investigate the presence of endoplasmic reticulum proteins (ER) or lipoproteins co-enriched in the EV preparations, anti-Calnexin (ab133615) and anti-Apolipoprotein A I (ab227455) was used, respectively. Appropriate secondary antibodies were used, and detection conducted using enhanced chemiluminescence reagent (Genesee Scientific, San Diego, CA).

### Cell lines propagation, treatment and western Blot

Human Intestinal Caco-2 and monocyte THP-1 cell lines were obtained from ATCC (Gaithersburg, MD, USA). Caco-2 cells were cultured at 37 °C in Eagle’s minimum essential medium (EMEM), supplemented with 15% heat inactivated fetal bovine serum (FBS) (Invitrogen), 2% of penicillin and streptomycin solution containing 10,000 units of penicillin and 10 mg of streptomycin/ml (Sigma-Aldrich, Saint Louis, MO) in a humidified atmosphere (5% CO2 and 95% air). For the experiments, 1 × 10^6^ Caco-2 cells per well were seeded in 6-well plates. The cells were treated with vehicle control (10 uL of EMEM media), 2% of defatted MOM (v/v) in EMEM, 2% defatted PMOM, 2% MOM supernatant (EVs free), 2% PMOM supernatant (exosome free), 10^10^ MOM_EV_ in EMEM, or 10^10^ PMOM_EV_. After 24 h of incubation, culture supernatants were collected for IL-8 (BD OptEIA™) by enzyme-linked immunosorbent assay (ELISA) and the cells collected for RNA extractions and/or protein extractions. THP-1 Cells were cultured in RPMI 1640 medium supplemented with 10,000 units of penicillin, 10 mg of streptomycin, and 10% heat inactivated FBS. For the experiments, THP-1 cells were seeded in 6-well plates at 1 × 10^6^ cells/well and activated by adding 100 nM phorbol 12-myristate-13-acetate (PMA), for 48 h at 37 °C. The cells were treated with 10^10^/mL of MOM_EV_ or PMOM_EV_ for 6 h. Supernatants and cells were processed as indicated above. For protein extractions, RIPA buffer containing 150 mM NaCl, 50 mM Tris (pH 8), 1% Triton X-100, and 0.1% sodium dodecyl sulfate (SDS), with Halt™ protease inhibitor cocktail (Thermo Fisher, Waltham, MA, USA) was used. The cell homogenates were centrifuged at 12,000 g for 10 min, at 4 °C and the protein concentration was measured using Pierce™ BCA Protein Assay Kit (Thermo Fisher Scientific, Waltham, USA). For western blots, primary antibodies against AKT (#9272), pAKT-T308 (#13038), pAKT-S473 (#9271), STAT3 (#4904), pSTAT3-S727 (#94994), p44/42 MAPK (Erk ½ #4695), pp44/42 MAPK (Erk1/2)-Thr202/Tyr204 (#9101), IGF-1R (#9750S) from Cell Signaling Technology, and IkB-alpha (MAB4299 R&D SYTEMS) were used. Anti-GAPDH (ab9485 abcam) and anti-βactin (#8457 Cell Signaling Technology) were used as loading controls. All the experiments were performed with biological and technical triplicates. Data was analyzed using ANOVA, and Tukey’s ‘Honest Significant Difference’ method was used to assign statistical significance for a *p* < 0.05. All the graphics and statistical analysis were generated using RStudio^61^.

### qRT-PCR and mRNA expression

RNA was isolated from cell lines using RNeasy® Mini Kit following the manufacturer’s protocol (QIAGEN, Germantown, MD). DNA was removed by treatment with DNase (QIAGEN, Germantown, MD) according to the manufacturer’s protocol. RNA quality was monitored on 1% agarose gels, and RNA quantification was performed using Thermo Scientific Nanodrop One Microvolume UV–vis spectrophotometer (Thermo Fisher Scientific, Grand Island, NY). qRT-PCR was performed as described^66^. Primer sequences used to determine relative transcript abundance are listed in Suppl. Table 3.

## Supplementary Information

Supplementary Information.

## Data Availability

miRNA raw data generated in this study was deposited in the NCBI Sequence Read Archive (SRA) under BioProject ID PRJNA930463. GEO accession GSE225840 https://www.ncbi.nlm.nih.gov/geo/query/acc.cgi?acc=GSE225840. Token yxwhykosdbgjxyp.
