# Supplementary material for: Pasteurization of human milk affects the miRNA cargo of EVs decreasing its immunomodulatory activity
Source: Sci Rep. 2023 Jun 21;13:10057. doi: 10.1038/s41598-023-37310-x (PMC10284810; doi:10.1038/s41598-023-37310-x)

Supplementary Information

Tables

Supplementary Table 1. miRNA Identified in Human milk EVs. Positive fold change shows enriched miRNA in $PMOM_{EV}$, while negative fold changes show enriched miRNA in MOM_{EV}.

miRNA	logFC	adj.P.Val
hsa-let-7b-5p	4.8	2.85E-06
hsa-miR-29c-5p	-4.5	8.79E-04
hsa-miR-1307-3p	-4.4	4.54E-04
hsa-miR-335-3p	-4.4	2.51E-03
hsa-miR-502-3p	-3.9	1.86E-03
hsa-miR-181a-2-3p	-3.8	2.98E-03
hsa-miR-452-5p	-3.8	2.63E-03
hsa-miR-205-5p	-3.8	3.03E-04
hsa-let-7a-5p	3.7	4.54E-04
hsa-miR-99a-3p	-3.7	3.84E-03
hsa-let-7a-5p	3.7	4.54E-04
hsa-miR-365a-3p	-3.6	4.54E-04
hsa-miR-365b-3p	-3.6	4.54E-04
hsa-miR-200a-5p	-3.5	7.54E-03
hsa-miR-374b-5p	-3.4	2.41E-03
hsa-miR-501-3p	-3.4	8.27E-03
hsa-miR-1307-5p	-3.4	5.41E-03
hsa-miR-23b-5p	-3.3	6.83E-03
hsa-miR-342-3p	3.3	4.54E-04
hsa-miR-106b-3p	-3.3	1.07E-02
hsa-miR-16-5p	3.3	6.30E-04
hsa-miR-194-5p	-3.3	7.54E-03
hsa-miR-29b-3p	-3.2	1.07E-02
hsa-miR-361-3p	-3.2	2.63E-03
hsa-miR-141-3p	-3.1	2.63E-03
hsa-miR-331-5p	-3.0	9.45E-03
hsa-miR-151a-3p	-3.0	9.31E-03
hsa-miR-146b-3p	-3.0	1.23E-02
hsa-miR-28-5p	-2.9	9.31E-03

hsa-miR-885-3p	-2.9	9.31E-03
hsa-let-7c	2.9	6.81E-03
hsa-let-7d-5p	2.9	6.57E-03
hsa-miR-193a-5p	2.9	3.84E-03
hsa-miR-148a-5p	-2.8	1.25E-02
hsa-miR-29c-3p	-2.8	1.25E-02
hsa-miR-660-5p	-2.7	2.24E-02
hsa-miR-128	-2.6	1.54E-02
hsa-miR-128	-2.6	1.54E-02
hsa-miR-32-5p	-2.6	1.57E-02
hsa-miR-93-5p	-2.6	9.31E-03
hsa-miR-20a-5p	-2.5	9.31E-03
hsa-miR-195-5p	2.5	9.31E-03
hsa-miR-98	-2.5	2.24E-02
hsa-miR-200b-3p	-2.5	3.07E-02
hsa-miR-92b-3p	-2.4	3.64E-02
hsa-miR-96-5p	-2.4	2.51E-02
hsa-miR-26a-5p	2.4	1.06E-02
hsa-miR-26a-5p	2.4	1.07E-02
hsa-miR-203	-2.4	2.60E-02
hsa-miR-99b-5p	-2.4	1.25E-02
hsa-miR-1292	-2.4	2.99E-02
hsa-miR-93-3p	-2.4	3.34E-02
hsa-miR-196a-5p	-2.3	3.07E-02
hsa-miR-187-3p	-2.3	3.60E-02
hsa-miR-17-3p	-2.3	3.08E-02
hsa-miR-196a-5p	-2.2	3.41E-02
hsa-miR-1301	-2.2	4.42E-02
hsa-miR-378c	-2.2	1.44E-02
hsa-miR-324-3p	-2.2	3.60E-02
hsa-miR-125a-5p	2.2	2.14E-02
hsa-miR-423-5p	2.1	9.45E-03
hsa-miR-146b-5p	2.1	1.35E-02
hsa-miR-103a-3p	2.1	3.41E-02
hsa-miR-103a-3p	2.1	3.52E-02

hsa-let-7g-5p	2.1	1.25E-02
hsa-miR-423-3p	2.0	3.87E-02
hsa-let-7b-3p	-2.0	7.11E-02
hsa-miR-629-5p	-2.0	5.01E-02
hsa-miR-107	2.0	4.32E-02
hsa-let-7f-5p	2.0	3.95E-02
hsa-let-7f-5p	2.0	3.99E-02
hsa-miR-106b-5p	-1.9	3.69E-02
hsa-miR-146a-5p	1.9	4.10E-02
hsa-miR-99b-3p	-1.8	7.38E-02
hsa-miR-22-3p	1.8	3.92E-02
hsa-miR-10a-5p	-1.7	8.51E-02
hsa-miR-671-3p	-1.7	8.51E-02
hsa-miR-200a-3p	-1.7	3.08E-02
hsa-miR-191-5p	1.7	1.07E-02
hsa-miR-378a-3p	1.6	4.10E-02
hsa-miR-3615	-1.6	1.14E-01
hsa-miR-339-3p	-1.6	1.11E-01
hsa-miR-21-5p	1.5	9.51E-02
hsa-miR-197-3p	1.5	1.11E-01
hsa-miR-182-5p	1.5	1.27E-01
hsa-miR-484	1.5	5.24E-02
hsa-miR-223-3p	1.4	1.21E-01
hsa-miR-24-3p	1.4	1.11E-01
hsa-miR-24-3p	1.4	1.11E-01
hsa-miR-23b-3p	-1.4	1.11E-01
hsa-miR-30e-5p	-1.4	1.22E-01
hsa-miR-30b-5p	-1.4	5.24E-02
hsa-miR-99a-5p	-1.4	1.21E-01
hsa-miR-671-5p	-1.4	1.74E-01
hsa-miR-320a	1.3	4.16E-02
hsa-miR-885-5p	1.3	1.08E-01
hsa-miR-320d	-1.3	2.19E-01
hsa-miR-181b-5p	-1.2	2.20E-01
hsa-miR-181b-5p	-1.2	2.20E-01

hsa-miR-181a-5p	-1.2	1.88E-01
hsa-miR-181a-5p	-1.2	1.88E-01
hsa-miR-23a-3p	-1.2	1.15E-01
hsa-let-7d-3p	-1.2	2.20E-01
hsa-miR-590-5p	1.2	2.52E-01
hsa-miR-148a-3p	1.1	1.11E-01
hsa-miR-320d	-1.1	3.05E-01
hsa-miR-200c-3p	-1.0	2.19E-01
hsa-miR-200b-5p	-1.0	3.51E-01
hsa-miR-34a-5p	-1.0	3.28E-01
hsa-miR-148b-3p	-1.0	3.28E-01
hsa-miR-497-5p	-0.9	3.61E-01
hsa-miR-30a-3p	-0.8	4.71E-01
hsa-miR-186-5p	-0.8	4.82E-01
hsa-miR-326	-0.8	5.12E-01
hsa-miR-574-3p	0.7	5.45E-01
hsa-miR-425-5p	0.7	5.20E-01
hsa-let-7i-5p	0.7	5.58E-01
hsa-miR-22-5p	-0.7	5.34E-01
hsa-miR-27a-3p	0.7	5.37E-01
hsa-miR-130a-3p	-0.6	5.58E-01
hsa-miR-664-5p	-0.6	6.18E-01
hsa-miR-30c-5p	-0.6	5.06E-01
hsa-miR-421	-0.6	6.44E-01
hsa-miR-30c-5p	-0.5	5.55E-01
hsa-miR-27b-3p	-0.5	6.44E-01
hsa-miR-151a-5p	0.5	6.44E-01
hsa-miR-26b-5p	0.5	6.64E-01
hsa-miR-30a-5p	0.5	5.72E-01
hsa-miR-192-5p	-0.5	6.87E-01
hsa-miR-877-5p	-0.5	6.75E-01
hsa-miR-15a-5p	0.4	6.92E-01
hsa-miR-652-3p	0.4	7.16E-01
hsa-miR-320c	0.4	7.06E-01
hsa-miR-92a-3p	0.4	6.62E-01

hsa-miR-185-5p	-0.4	6.97E-01
hsa-miR-92a-3p	0.4	6.69E-01
hsa-miR-320b	0.4	6.96E-01
hsa-miR-324-5p	-0.4	7.48E-01
hsa-miR-320c	0.4	7.69E-01
hsa-miR-28-3p	-0.4	7.69E-01
hsa-miR-222-3p	-0.4	7.80E-01
hsa-miR-193b-5p	0.3	7.72E-01
hsa-let-7e-5p	0.3	7.72E-01
hsa-miR-224-5p	-0.3	7.56E-01
hsa-miR-335-5p	0.3	6.87E-01
hsa-miR-345-5p	0.3	8.07E-01
hsa-miR-152	-0.3	8.07E-01
hsa-miR-125a-3p	-0.3	8.07E-01
hsa-miR-19a-3p	-0.3	7.72E-01
hsa-miR-125b-5p	0.2	8.07E-01
hsa-miR-25-3p	-0.2	8.39E-01
hsa-miR-125b-5p	0.2	8.31E-01
hsa-miR-1287	-0.2	8.82E-01
hsa-miR-2110	-0.2	8.84E-01
hsa-miR-375	-0.2	8.87E-01
hsa-miR-320b	0.1	8.84E-01
hsa-miR-30d-5p	0.1	8.84E-01
hsa-miR-130b-3p	0.1	9.10E-01
hsa-miR-193b-3p	-0.1	9.10E-01
hsa-miR-183-5p	0.1	9.37E-01
hsa-miR-221-3p	0.1	9.37E-01
hsa-miR-29a-3p	-0.1	9.38E-01
hsa-miR-19b-3p	0.0	9.73E-01
hsa-miR-19b-3p	0.0	9.73E-01

Supplementary Table 2. mRNA Targets of miRNA identified in MOM_{EV} and $PMOM_{EV}$ using IPA software. Positive fold change shows enriched miRNA in $PMOM_{EV}$, while negative fold changes show enriched miRNA in MOM_{EV}. With “*” are denoted the miRNA enriched in $PMOM_{EV}$.

ID	Symbol	Fold Change	adj.P.Val	Count of targets	Target mRNA	Pathways
hsa-miR-1307-3p	miR-1307-3p (miRNAs w/seed CUCGGCG)	-4.413	4.54E-04	1	PRKCZ	-
hsa-miR-1307-5p	miR-1307-5p (miRNAs w/seed CGACCGG)	-3.374	5.41E-03	1	PRKCE	-
hsa-miR-141-3p	miR-141-3p (and other miRNAs w/seed AACACUG)	-3.116	2.63E-03	7	**CD80**, LPAR3, **MAP2K4**, NME1, P2RY1, PRKACB, SELE	T Cell Receptor signaling, STAT3 pathway, IL-15 production, AMPK signaling, NF-kB signaling
hsa-miR-151a-3p	miR-151-3p (and other miRNAs w/seed UAGACUG)	-3.02	9.31E-03	1	HTR1F	-
hsa-miR-93-5p	miR-17-5p (and other miRNAs w/seed AAAGUGC)	-2.57	9.31E-03	8	**CDKN1A**,MAP3K12, MAP3K2, MASTL, PAK5, **PFKP**, S1PR1, **TGFBR2**, **TLR7**, TNFRSF21, BMPR2	AMPK signaling, NF-kB signaling, STAT3 pathway
hsa-miR-181a-2-3p	miR-181a-2-3p (and other miRNAs w/seed CCACUGA)	-3.763	2.98E-03	7	CD3G, GPR12, GRK4, HTR2C, HTR5A, NPFFR1, NPR3	-
hsa-miR-194-5p	miR-194-5p (miRNAs w/seed GUAACAG)	-3.258	7.54E-03	1	**PRKAR1A**	AMPK signaling
hsa-miR-205-5p	miR-205-5p (and other miRNAs w/seed CCUUCAU)	-3.752	3.03E-04	5	CALCRL, CCR8, LRP1,	IL-15 production

					PRKCE, **YES1**	
hsa-miR-23b-5p	miR-23a-5p (and other miRNAs w/seed GGGUUCC)	-3.299	3.03E-04	11	**CKM**, **CSNK2B**, CTLA4, HCST, JMJD6, **MAPK13**, P2RY4, PHKG1, STK38, **TEC**, TLR4	AMPK signaling, NF-kB signaling, T Cell receptor signaling, STAT3 pathway, IL-15 production
hsa-miR-194-5p	miR-194-5p (miRNAs w/seed GUAACAG)	-3.258	7.54E-03	2	FZD6, NME1	-
hsa-miR-29c-5p	miR-29c-5p (miRNAs w/seed GACCGAU)	-4.465	8.79E-04	1	FZD3	-
hsa-miR-331-5p	miR-331-5p (and other miRNAs w/seed UAGGUAU)	-3.03	9.45E-03	6	LGR4, **LYN**, **PFKFB3**, **PFKP**, TAS2R30, ULBP2	IL-15 production, AMPK signaling
hsa-miR-335-3p	miR-335-3p (miRNAs w/seed UUUUCAU)	-4.36	2.51E-03	3	**EPHA3**, **TGFBR1**, **TGFBR2**	IL-15 production, NF-kB signaling, STAT3 pathway
hsa-miR-361-3p	miR-361-3p (miRNAs w/seed CCCCCAG)	-3.172	2.63E-03	21	ADGRG3, CAMK2B, CAMKK1, **CD40**, CD79A, CLSR3, CXCR5, GPR37L1, **IL1RL2**, **IL21R**, LIMK2, **MAP3K10**,	NF-kB signaling, STAT3 pathway

					MEX3, MKNK2, MTNR1B, P2RY2, PIP4K2C, PTAFR, RHO, RPS6KB2, STR5	
hsa-miR-365b-3p	miR-365-3p (and other miRNAs w/seed AAUGCCC)	-3.563	4.54E-04	9	ACVR1, **CDKN1A**, HTR1F, IL1RAP, LPAR5, P2RY1, **PIK3R3**, SGK1, **TBK1**	NF-kB signaling, AMPK signaling, STAT3 pathway, T Cell receptor signaling
hsa-miR-374b-5p	miR-374b-5p (and other miRNAs w/seed UAUAAUA)	-3.429	2.41E-03	2	FZD3, **NCK1**	T Cell Receptor signaling
hsa-miR-452-5p	miR-452-5p (and other miRNAs w/seed ACUGUUU)	-3.756	2.63E-03	1	**MAP4K4**	NF-kB signaling
hsa-miR-502-3p	miR-501-3p (and other miRNAs w/seed AUGCACC)	-3.907	2.63E-03	4	CCR1, CDK6, FCGR1A, **ITK**	T Cell Receptor signaling, IL-15 production
hsa-miR-28-5p	miR-708-5p (and other miRNAs w/seed AGGAGCU)	-2.946	9.31E-03	12	AGTR2, **CDKN1A**, CNTFR, GPBAR1, GPR139, **IKBKB**, MPL, NPY2R, PDK4, RHO, S1PR1, AS2R14	AMPK signaling, STAT3 pathway, T Cell Receptor signaling, NF-kB signaling
hsa-miR-885-3p	miR-885-3p (miRNAs w/seed GGCAGCG)	-2.93	9.31E-03	8	ADORA1, **AKT2**, CD79A, CDK4,	AMPK signaling, T Cell Receptor signaling, NF-

					GPR173, **NTRK2**, PLXNA1, PLXNB1	kB signaling, STAT3 pathway, IL-15 production
hsa-miR-16-5p*	miR-16-5p (and other miRNAs w/seed AGCAGCA)	3.262	6.30E-04	26	ACVR2B, AK4, **AKT3**, CCND3, **CD28**, **CD40**, **CD80**, CDK5R1, CDK6, CHEK1, CRKL, **EGFR**, **FGFR1**, GABBR1, GPR171, GPR63, HLA-DQB2, HTR2A, HTR4, **IGF1R**, IGFR2, PHKB, PLK1, **RAF1**, RPS6KA3, SGK1, **MAP2K1**, **MAP2K4**, **MAPK3**	AMPK signaling, T Cell Receptor Signaling, NF-kB signaling, STAT3 pathway, IL-15 Production
hsa-miR-193a-5p*	miR-193a-5p (miRNAs w/seed GGGUCUU)	2.861	3.84E-03	8	ACVR1, FCER2, **HLA-F**, **IL2RG**, KLRD1, **MTOR**, NCR1, **PIK3R3**	T Cell Receptor Signaling, STAT3 pathway, AMPK signaling
hsa-miR-342-3p*	miR-342-3p (miRNAs w/seed CUCACAC)	3.281	4.54E-04	1	HTR2C	-
hsa-miR-423-5p*	miR-423-5p (and other miRNAs	2.148	2.41E-03	27	ACVR1B,	AMPK signaling,

	w/seed GAGGGGC)				ADGRB2, ADGRL1, **CDKN1A**, CELSR2, **CHRND**, CXCR5, GLP1R, GPR173, GPR20, GRM4, HRH2, LIMK1, **MAPK3**, MAPK4, MKNK2, **NGFR**, NPFR1, NPR3, NRP1, NTSR1, **PFKFB3**, **PRKACA**, **PRKAR1B**, PTGFR, PTK2B, RPS6KA4	STAT3 pathway, T Cell signaling, NF-kB signaling
hsa-let-7b-5p*	let-7a-5p (and other miRNAs w/seed GAGGUAG)	4.805	2.85E-06	28	ACVR1C, ADGRG1, **ADRB2**, **ADRB3**, AURKB, CCR7, CDK6, **CDKN1A**, **CHRNA7**, CSNK1D, DRD3, FAS, FZD3, GPR26,	AMPK signaling, T Cell Receptor Signaling, NF-kB signaling, STAT3 pathway, IL-15 Production

					GPR63, HTR1E, **ICOS**, **IGF1R**, ITGB3, MAP3K13, MAPK6, **PRKAR2A**, PTAFR, **TGFBR1**, **TGFBR2**, **TGFBR3**, **TLR4**, XCR1	

Supplementary Table 3. List of primers used on qRT-PCR assays.

Gene	Forward Sequence (5' to 3')	Reverse Sequence (5' to 3')
GAPDH	GAAGGTGAAGGTCGGAGTC	GAAGATGGTGATGGGATTTC
AKT	ATGAGCGACGTGGCTATTGTGAAG	GAGGCCGTCAGCCACAGTCTGGATG
IGF1R	AGGATATTGGGCTTTACAACCTG	GAGGTAACAGAGGTCAGCATTTT
STAT3	TGGAGCTGCGGCAGTTTCTG	CCGCATCTGGTCCAGCGCAG
IL10	GAGAGAAGCTGAAGACCCTCTG	TCATTCATGGCCTTGTAGACAC
TNFα	GCCCACGTCGTAGCAA	GTCTTTGAGATCCATGCCAT
IL1β	GAGCTGAAAGCTCTCCACCT	TTCCATCTTCTTCTTTGGGT
IL15	GGATTTACCGTGGCTTTGAGTAATGAG	GAATCAATTGCAATCAAGAAGTG
OAS2	ACCCGAACAGTTCCCCCTGGT	ACAAGGGTACCATCGGAGTTGCC
RIG1	TAAGGGGATGATGGCAGGTG	TGGGCCAGTTTTCCTTGTCT
INFα	TGCTTTACTGATGGTCCTGGT	TCATGTCTGTCCATCAGACAG
IL29	GGACGCCTTGGAAGAGTCACT	AGAAGCCTCAGGTCCCAATTC

Supplementary Figures

Supplementary Figure1. EVs Physical Characterization and Quantification. (A) Nanoparticle tracking analysis showing size and particles number distribution obtained from MOM (left panel) and PMOM (right panel). **(B)** Zeta potential diagram for MOM_{EV} and $PMOM_{EV}$.

A

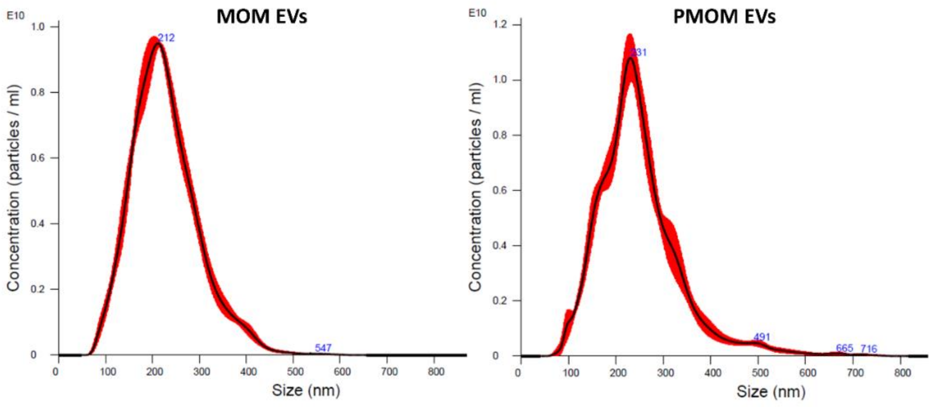
MOM EVs
PMOM EVs
E10
Concentration (particles / ml)
Size (nm)
212
547
231
491
665
716

B

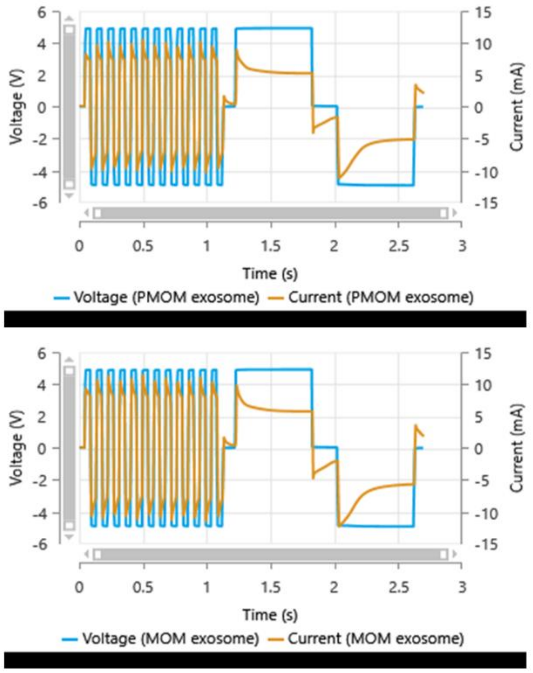
Voltage (V)
Current (mA)
Time (s)
Voltage (PMOM exosome)
Current (PMOM exosome)
Voltage (MOM exosome)
Current (MOM exosome)

Supplementary Figure 2. Protein levels in Caco2 cells are not affected after treatment with human milk EVs. Western blotting showing total **(A)** AKT, **(B)** AKT phosphorylation S-473, **(C)** AKT phosphorylation T-308, **(D)** corresponding blotting images and **(E)** total STAT3, **(F)** STATS3 phosphorylation S-727, **(G)** total IGF protein levels and **(H)** corresponding blotting images. GAPDH was used as a loading control. Two biological replicates are shown for each treatment as indicated. Quantification of band intensity was performed with ImageJ.

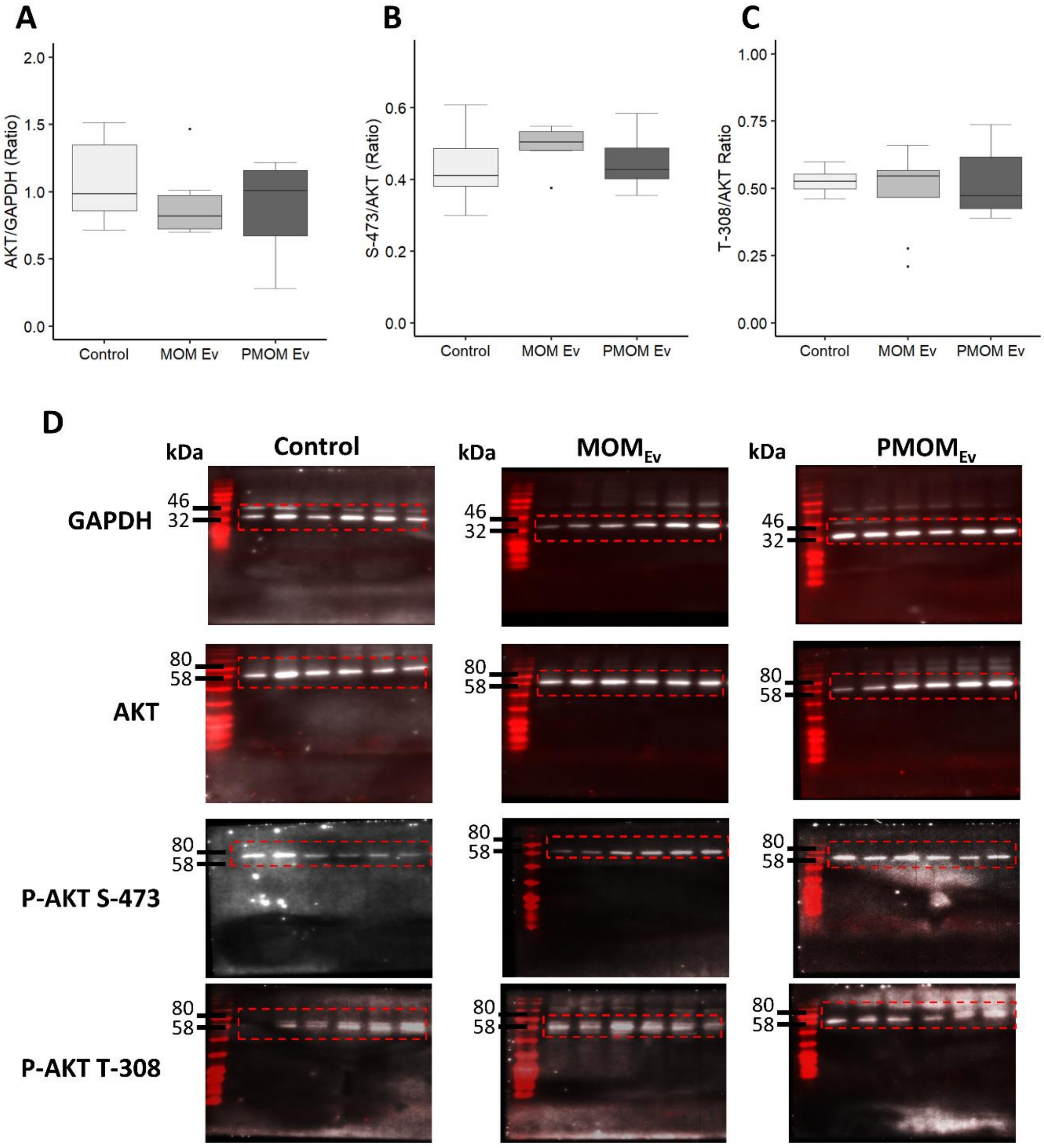

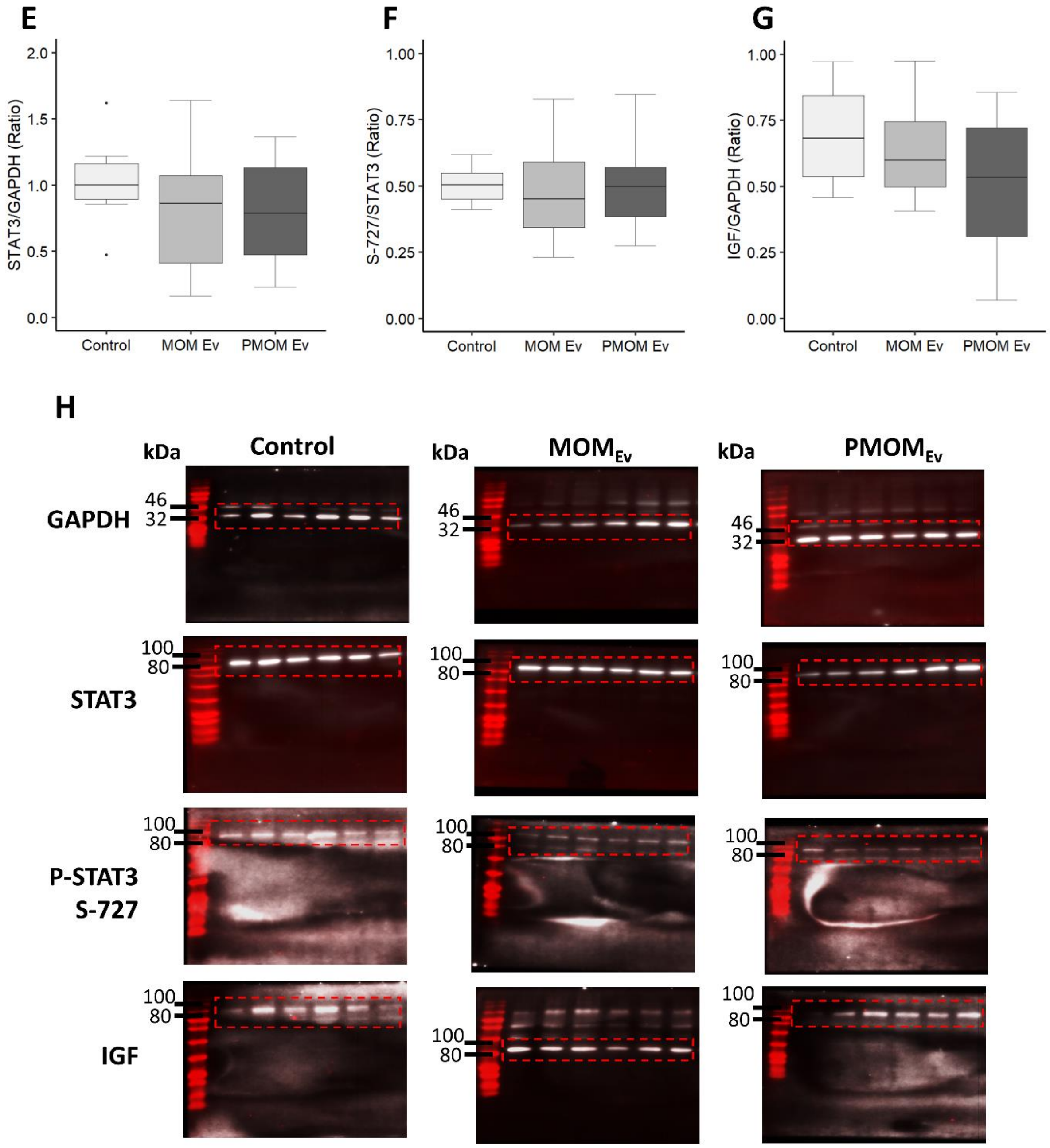

Supplementary Figure 3. Pasteurization affects the detection of endocytic EV markers. Extended Figure 1. Western blot analyses were performed on total protein extractions of MOM$_{EV}$ and PMOM$_{EV}$ enriched fractions using anti-CD63, anti-CD9, as well as anti-Calnexin as a negative control and anti-ApoA1 as a lipoprotein marker. Red dashed lines delimit the cropped area.

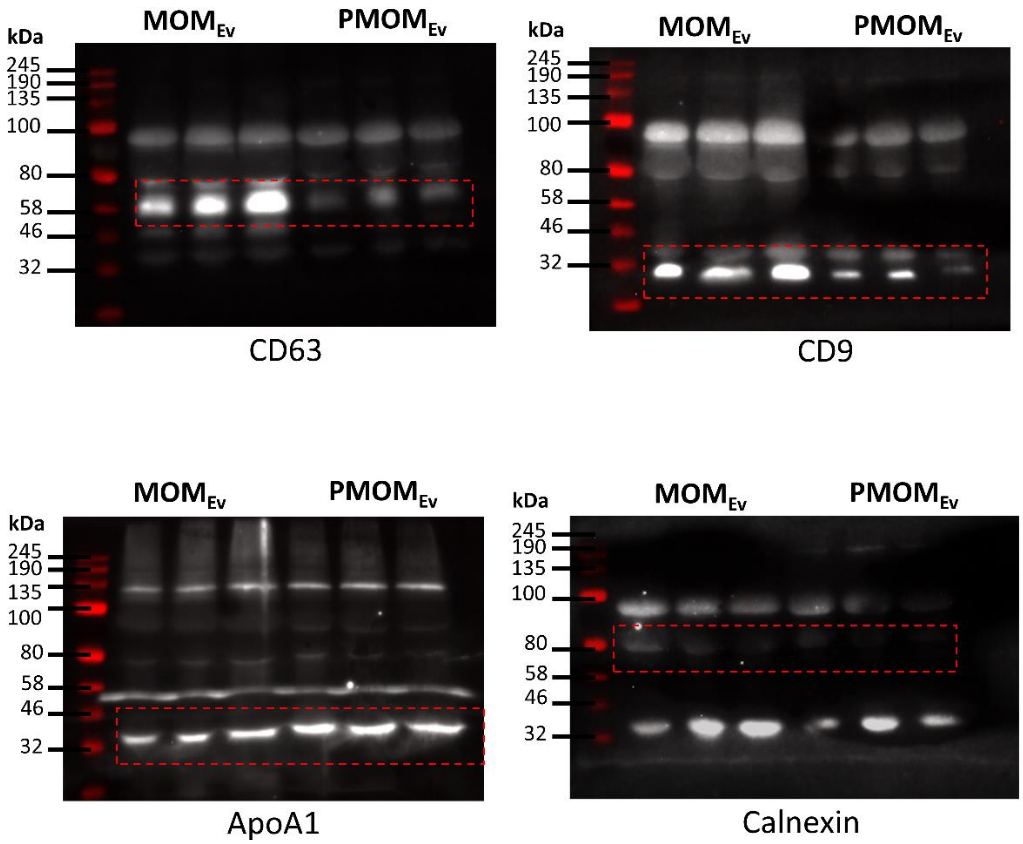

Supplementary Figure 4. Lipoproteins do not stimulate gene expression of master regulator IL15 in THP-1 macrophages. The relative expressions of IL-15 are shown as fold change relative to the vehicle control. The experiments were performed with biological and technical triplicates. Single way ANOVA and mean comparisons were performed, different letters indicate statistical significance at $p < 0.05$.

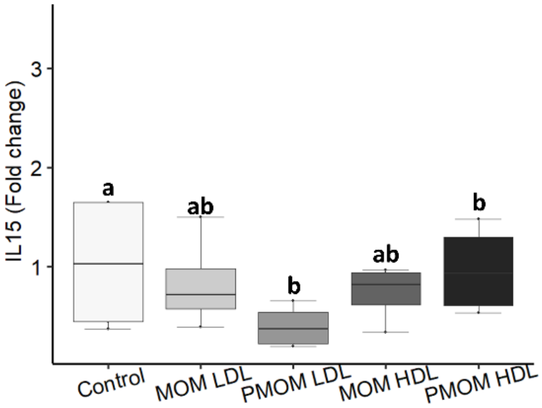

Supplementary Figure 5. Pasteurization affects the Effect of MOMEV in AMP, IKBα and Stat3 signaling pathways. Extended Figure 8. Western blot analyses were performed on total protein extractions THP1 macrophages treated with MOM_{EV} and $PMOM_{EV}$ enriched fractions using anti-β-actin, anti-IKBα, anti-STAT3, anti-P-STAT3-Ser727, anti-ERK and anti-P-ERK-Thr202/Tyr204. Red dashed lines delimit the cropped area.

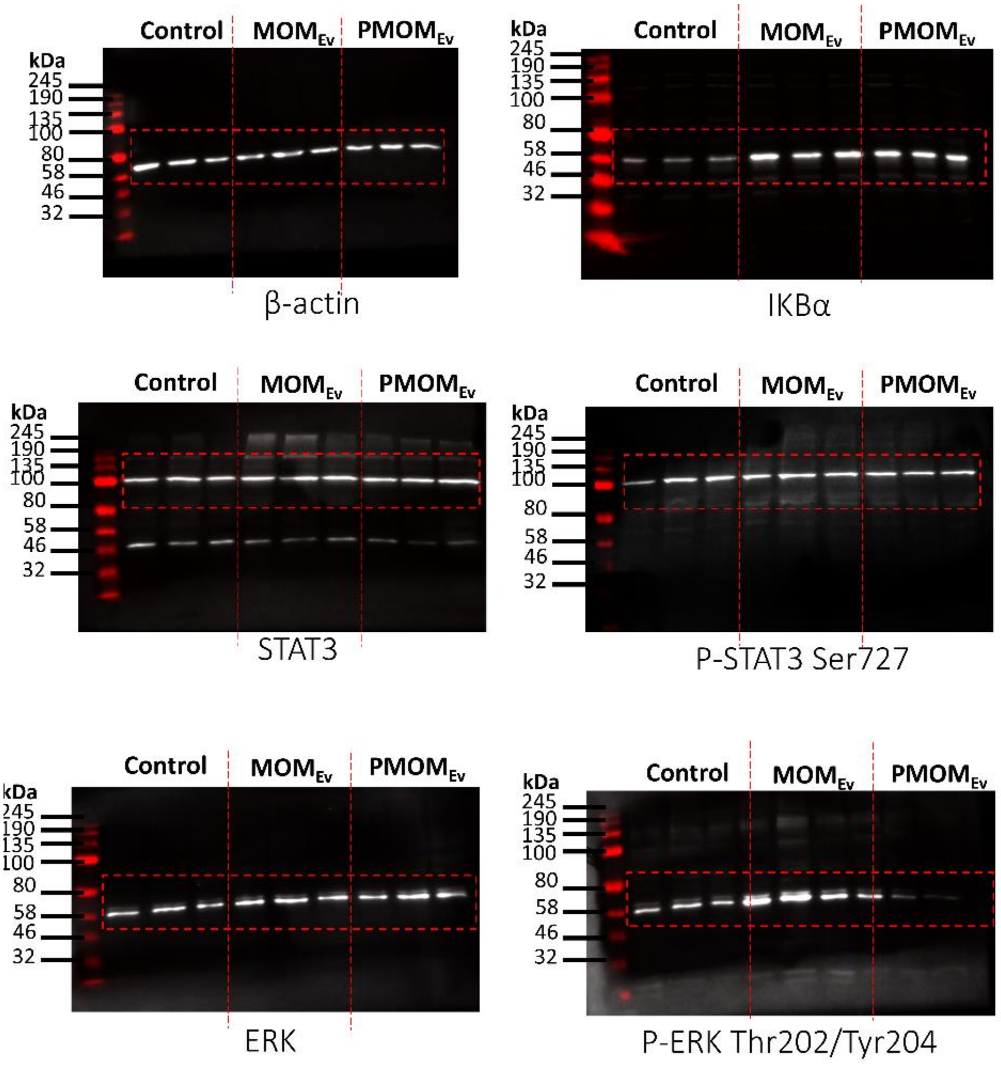

Supplement: Supplementary file 1 — Supplementary Information. [file 41598_2023_37310_MOESM1_ESM.pdf]
